# Fusion anomaly of the pancreatic tail and spleen: a case report

**DOI:** 10.1186/s13256-017-1391-3

**Published:** 2017-08-27

**Authors:** Ahmad Khalid Omeri, Shunro Matsumoto, Maki Kiyonaga, Ryo Takaji, Yasunari Yamada, Yumiko Ando, Hiromu Mori, Hiroki Uchida, Yukio Iwashita, Masayuki Ohta, Masafumi Inomata

**Affiliations:** 1 Department of Radiology, Faculty of Medicine, Oita University, 1-1 Idaigaoka, Hasama-machi, Yufu city, 879-5593 Oita Japan; 2 Department of Gastroenterological and Pediatric Surgery, Faculty of Medicine, Oita University, Yufu, Oita 879-5593 Japan

**Keywords:** Pancreas, Spleen, Multidetector computed tomography, Magnetic resonance imaging, Anomaly, Splenopancreatic fusion

## Abstract

**Background:**

Splenopancreatic fusion is a rare anomaly that is often associated with trisomy 13. Its diagnosis can be important in patients scheduled to undergo distal pancreatectomy or splenectomy, to avoid possible intraoperative or postoperative complications.

**Case presentation:**

An 82-year-old Japanese man was referred to our hospital for further evaluation and treatment for a solitary hepatocellular carcinoma based on liver cirrhosis. Triple-phase contrast-enhanced multidetector-row computed tomography and magnetic resonance imaging showed a splenopancreatic fusion as well as a solitary hepatocellular carcinoma in segment VIII of his liver.

**Conclusions:**

Fusion of the pancreatic tail and spleen is a rare and asymptomatic anomaly. Its detection can be improved by the use of multidetector computed tomography or magnetic resonance imaging.

## Background

Splenopancreatic fusion is an uncommon abnormality that is among the splenopancreatic field abnormalities associated with various congenital disorders, mainly trisomy 13 [1, 2]. Other splenopancreatic field abnormalities reported in the English language literature include ectopic pancreatic tissue in the spleen or accessory spleen, ectopic splenic tissue in the pancreas, as well fusion of the pancreatic tail and splenic hilum or accessory spleen [1–4]. While their pathological aspects have been described, only one case report has considered their radiological findings [5]. Here we report a case of splenopancreatic fusion in an 82-year-old man and discuss the imaging findings obtained using triple-phase contrast-enhanced multidetector-row computed tomography (3P-CE-MDCT) and contrast-enhanced magnetic resonance imaging (MRI).

## Case presentation

An 82-year-old Japanese man with a history of liver cirrhosis developed a solitary hepatocellular carcinoma (HCC) in segment VIII of his liver. He was referred to our hospital for further evaluation and treatment although he had no symptoms. He had no medical, family, or psychosocial history related to the disease, nor did he have a history or findings of congenital disorders. A physical examination revealed no significant findings. Laboratory tests showed elevated serum aspartate aminotransferase (0.83 μkat/L), alanine aminotransferase (0.99 μkat/L), and total bilirubin (20.35 μmol/L). Testing for the hepatitis C virus antibody was positive, and serum tumor markers for HCC were elevated: alpha-fetoprotein (AFP) 13.5 ng/mL, protein induced by vitamin K absence/antagonist-II (PIVKA-II) 68 mAU/mL. Other laboratory test results were within normal limits.

**Fig. 1:**
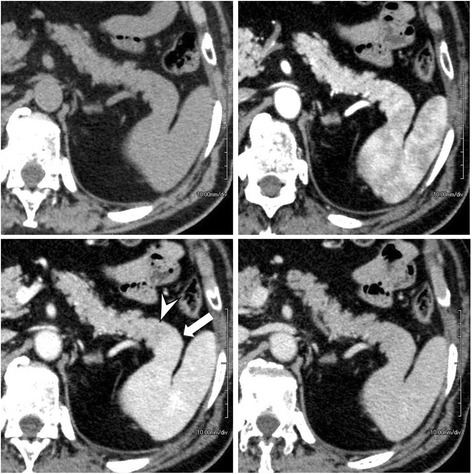
Axial triple-phase contrast-enhanced multidetector-row computed tomography images show the pancreatic tail in continuity with the spleen. The boundary between the organs is difficult to identify on unenhanced multidetector-row computed tomography (*upper left*); however, in the portal venous phase (*lower left*), the boundary between the pancreas (*arrowhead*) and spleen (*arrow*) is clearly visible

**Fig. 2:**
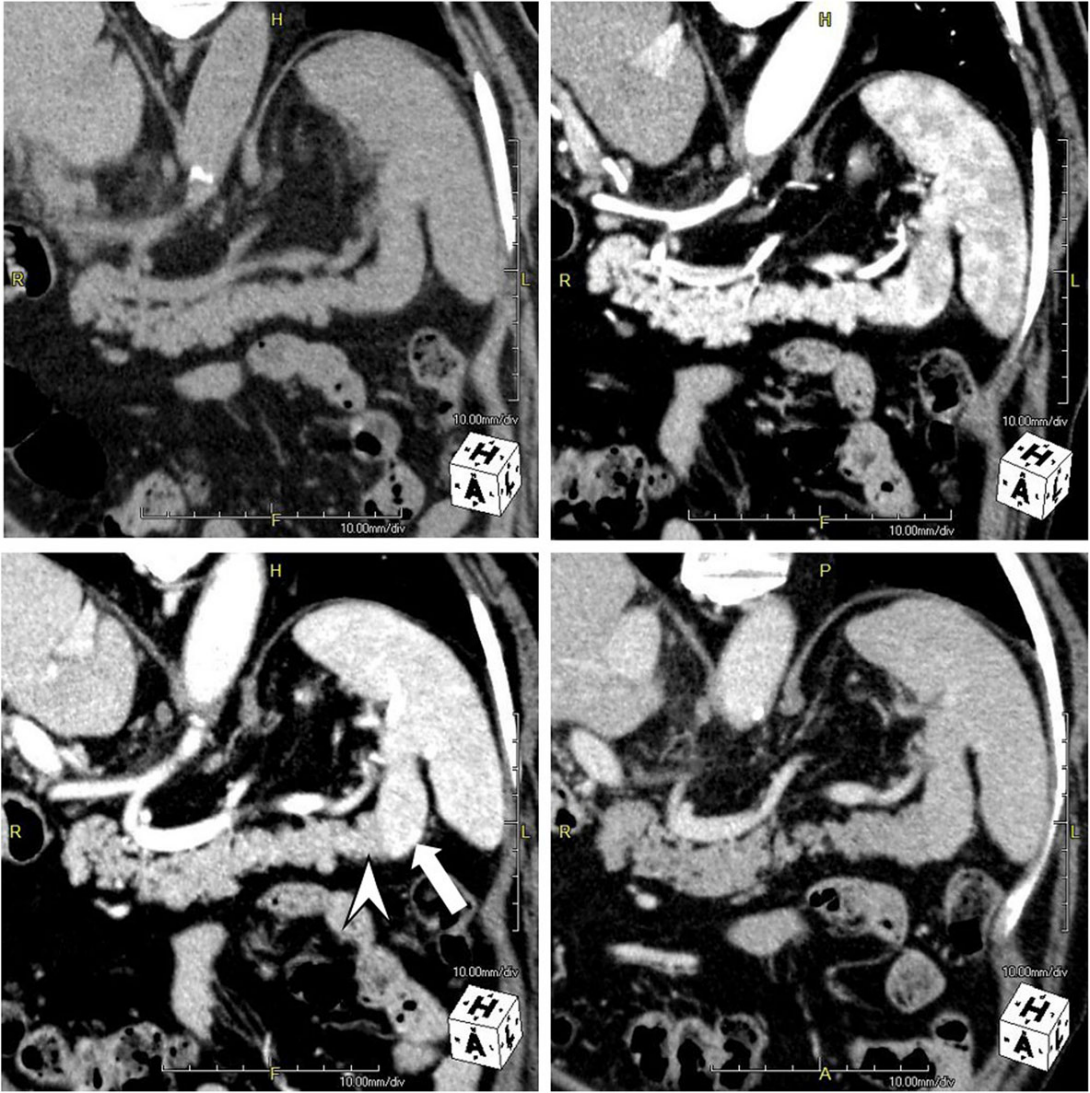
Coronal multiplanar reconstructed images of triple-phase contrast-enhanced multidetector-row computed tomography show fusion of the pancreatic tail (*arrowhead*) to the lower medial pole of the spleen (*arrow*), resulting in its bi-loaded configuration

**Fig. 3:**
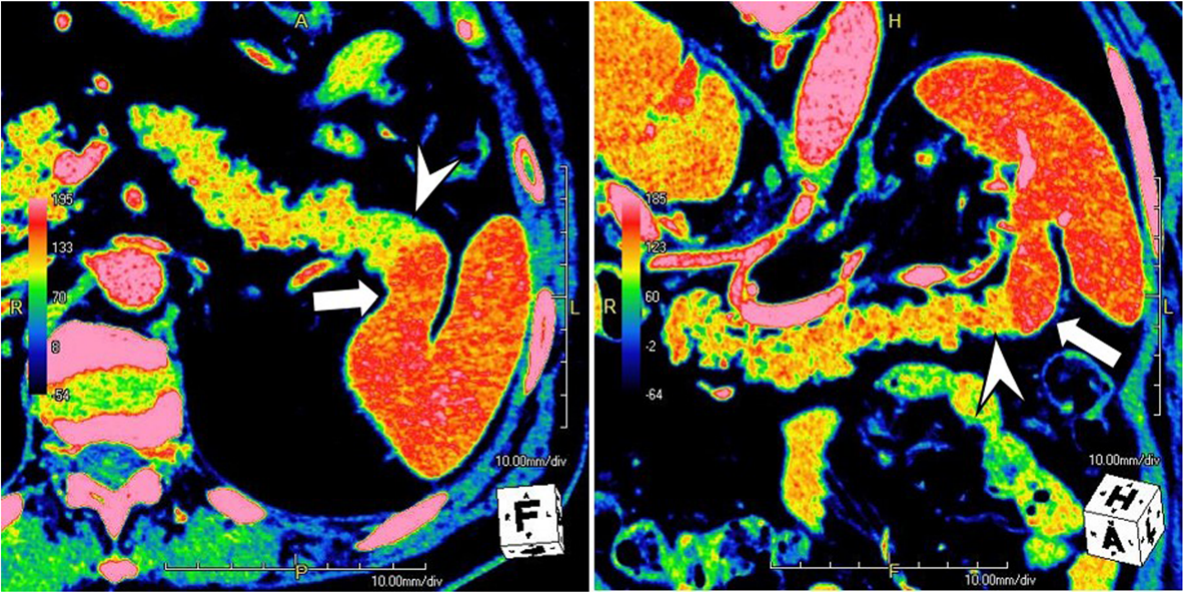
Colored maps of the axial and coronal multiplanar reconstructed images obtained in the portal venous phase easily differentiated the pancreas (*arrowhead*) and spleen (*arrow*) based on differences in their contrast enhancement patterns

Three days after admission, he underwent unenhanced multidetector-row computed tomography (MDCT) and 3P-CE-MDCT examinations. The former revealed a solitary hypodense mass in segment VIII of his liver and continuity of the pancreatic tail with the spleen, which was also noted on 3P-CE-MDCT images (Fig. 1). Curved multiplanar reconstructed images showed that the pancreatic tail was fused to the lower pole of his spleen (Fig. 2). The pancreatic tissue showed homogenous contrast enhancement during the arterial phase, and the splenic tissue the expected heterogeneous pattern. On the portal venous phase, the spleen became homogenous and intensely enhanced compared to the pancreatic tissue, with a distinct line of demarcation between the two organs. Both showed nearly similar contrast enhancement during the delayed phase. Colored maps of the axial and coronal images reformatted from the portal venous phase clearly depicted the fusion border and distinguished between splenic and pancreatic tissues (Fig. 3).

**Fig. 4:**
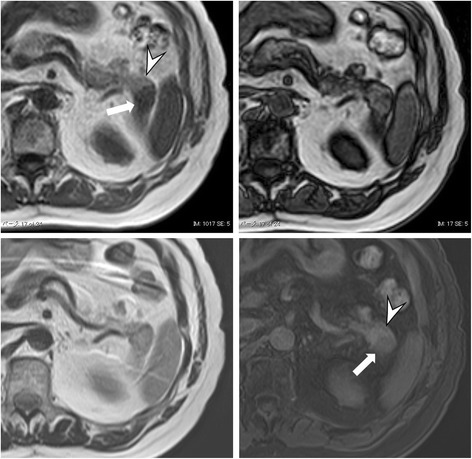
In-phase T1-weighted image (*upper left*) clearly shows the boundary between the pancreatic tail (*arrowhead*) and the spleen (*arrow*). On out-of-phase T1-weighted image (*upper right*), a fat plane between the pancreatic tail and splenic tissue could not be identified and thus suggested fusion. T2-weighted image could not identify the fused border. Fat-suppressed T1-weighted image after gadolinium-ethoxybenzyl-diethylenetriamine pentaacetic acid (*lower right*) differentiated between the two tissues based on their contrast differences

One week after the MDCT examination, he underwent MRI; in-phase and out-of-phase T1-weighted images (T1WI), T2-weighted images (T2WI), and dynamic contrast-enhanced fat suppressed T1WI were obtained. A fat plane between the pancreatic tail and splenic tissue could not be identified on out-of-phase T1WI, again suggestive of fusion. In-phase T1WI clearly distinguished the border between the two tissues based on the differences in signal intensity. However, on T2WI, the fusion border could not be easily identified. Fat-suppressed T1WI after gadolinium-ethoxybenzyl-diethylenetriamine pentaacetic acid (Gd-EOB-DTPA) administration showed a pattern of contrast enhancement similar to that seen on 3P-CE-MDCT (Fig. 4).

Our patient underwent laparoscopic liver resection for HCC in segment VIII. His postoperative course was uneventful and he was discharged 2 weeks after surgery. However, HCC recurrence was detected on follow-up CT performed 6 months after his discharge from our hospital. He was treated several times with transarterial chemo-embolization for recurrent HCC, but he died 2 years after the initial admission to our hospital, due to the progression of the tumor.

## Discussion

Fusion of the pancreatic tail and spleen reflects their disturbed embryogenesis, as the two organs are located in the dorsal mesoduodenum and dorsal mesogastrium, respectively, and are thus in close proximity [6, 7]. The pancreas is formed by the union of ventral and dorsal buds originating in two different regions in the distal foregut. The ventral bud, which arises from the hepatic diverticulum, develops into the inferior portion of the pancreatic head and uncinate process. The dorsal bud, which arises from the duodenum, develops into the superior portion of the head, body, and tail of the pancreas. The two primordia fuse after a 180° rotation of the duodenum at the end of 6th week of gestation. The head and body of the pancreas grow within the dorsal mesoduodenum and extend to the dorsal mesogastrium, whereas the tail of the pancreas lies within the dorsal mesogastrium [8–10].

The spleen arises as multiple mesenchymal buds that condense between the folds of the dorsal mesogastrium during the 5th week of gestation [7, 11]. During the 10th week, because of the counterclockwise rotation of the stomach and duodenum, the mesogastrium and mesoduodenum swing to the left, thus making a hairpin turn at the spleen [10] and positioning the pancreatic tail and spleen in the left upper quadrant. The splenic primordia coalesce to yield a single splenic mass by the end of the 12th week [7–9, 11]. During these processes, a disturbance in the embryogenesis of one or both organs may result in several anomalous conditions, including one or multiple accessory spleens around the pancreatic tail, intrapancreatic accessory spleen, and, as in our patient, fusion of the pancreatic tail and spleen [5, 9, 11–13]. We speculate that the development of one of the splenic buds in the lower dorsal mesogastrium is partially arrested by the pancreatic tail, located in the same region, such that, later on, the free surface of the splenic bud coalesces with the main splenic mass. This sequence of events is supported by the case reported by Balli *et al*. [5], in which, as in our patient, the pancreatic tail was fused to the medial aspect of the lower pole of the spleen.

Yang *et al.* [1] reported two cases of trisomy 13 associated with fusion of the pancreatic tail and spleen and concluded that splenopancreatic fusion suggests a diagnosis of trisomy 13. Gomi *et al.* [2] reviewed the macroscopic and microscopic findings of 21 individuals with trisomy 13 and compared them with those of 1060 controls without trisomy 13. Fusion of the pancreatic tail and splenic hilum and/or accessory spleen was noted in 17 of the 21 patients, versus only 2 of the 1060 controls. By contrast, Peres *et al.* [3] reported four cases of splenopancreatic fusion occurring together with other congenital anomalies, including trisomy 21, osteocraniostenosis syndrome, isolated congenital heart defect, and prune belly syndrome, but none of these patients had trisomy 13. The authors therefore concluded that splenopancreatic fusion should not be interpreted as pathognomonic of trisomy 13. Lehman *et al.* [4] also reported a case of splenopancreatic fusion associated with Schinzel–Giedion syndrome.

Our report includes a description of the 3P-CE-MDCT and MRI contrast enhancement patterns that characterized the splenopancreatic fusion in an otherwise developmentally normal patient. These radiological findings may assist radiologists in establishing the correct diagnosis and may prevent costly and unnecessary examinations. They are also important for surgeons treating patients scheduled to undergo distal pancreatectomy or splenectomy, to avoid possible intraoperative or postoperative complications such as bleeding or pancreatic ductal leaks.

## Conclusions

Splenopancreatic fusion is an uncommon, asymptomatic congenital abnormality, but the use of MDCT or MRI may increase the rate of its detection.

## Abbreviations

3P-CE-MDCT: Triple-phase contrast-enhanced multidetector-row computed tomography
CT: Computed tomography
HCC: Hepatocellular carcinoma
MDCT: Multidetector-row computed tomography
MRI: Magnetic resonance imaging
T1WI: T1-weighted images
T2WI: T2-weighted images
